# Effect of high-dose simvastatin on cognitive, neuropsychiatric, and health-related quality-of-life measures in secondary progressive multiple sclerosis: secondary analyses from the MS-STAT randomised, placebo-controlled trial

**DOI:** 10.1016/S1474-4422(17)30113-8

**Published:** 2017-08

**Authors:** Dennis Chan, Sophie Binks, Jennifer M Nicholas, Chris Frost, M Jorge Cardoso, Sebastien Ourselin, David Wilkie, Richard Nicholas, Jeremy Chataway

**Affiliations:** a Brighton and Sussex Medical School, Brighton, UK; b Department of Clinical Neurosciences, University of Cambridge, UK; c Brighton and Sussex University Hospitals NHS Trust, Brighton, UK; d London School of Hygiene and Tropical Medicine, London, UK; e Centre for Medical Image Computing, University College London, London, UK; f Queen Square Multiple Sclerosis Centre, Department of Neuroinflammation, UCL Institute of Neurology, University College London, London, UK; g Imperial College London, London, UK

## Abstract

**Background:**

In the 24-month MS-STAT phase 2 trial, we showed that high-dose simvastatin significantly reduced the annualised rate of whole brain atrophy in patients with secondary progressive multiple sclerosis (SPMS). We now describe the results of the MS-STAT cognitive substudy, in which we investigated the treatment effect on cognitive, neuropsychiatric, and health-related quality-of-life (HRQoL) outcome measures.

**Methods:**

We did a secondary analysis of MS-STAT, a 24-month, double-blind, controlled trial of patients with SPMS done at three neuroscience centres in the UK between Jan 28, 2008, and Nov 4, 2011. Patients were randomly assigned (1:1) to either 80 mg simvastatin (n=70) or placebo (n=70). The cognitive assessments done were the National Adult Reading Test, Wechsler Abbreviated Scale of Intelligence, Graded Naming Test, Birt Memory and Information Processing Battery (BMIPB), Visual Object and Space Perception battery (cube analysis), Frontal Assessment Battery (FAB), and Paced Auditory Serial Addition Test. Neuropsychiatric status was assessed using the Hamilton Depression Rating Scale and the Neuropsychiatric Inventory Questionnaire. HRQoL was assessed using the self-reported 36-Item Short Form Survey (SF-36) version 2. Assessments were done at study entry, 12 months, and 24 months. Patients, treating physicians, and outcome assessors were masked to treatment allocation. Analyses were by intention to treat. MS-STAT is registered with ClinicalTrials.gov, number NCT00647348.

**Findings:**

Baseline assessment revealed impairments in 60 (45%) of 133 patients on the test of frontal lobe function (FAB), and in between 13 (10%) and 43 (33%) of 130 patients in tests of non-verbal and verbal memory (BMIPB). Over the entire trial, we noted significant worsening on tests of verbal memory (T score decline of 5·7 points, 95% CI 3·6–7·8; p<0·0001) and non-verbal memory (decline of 6·8 points, 4·8–8·7; p<0·0001). At 24 months, the FAB score was 1·2 points higher in the simvastatin-treated group than in the placebo group (95% CI 0·2–2·3). The simvastatin group also had a 2·5 points better mean physical component score of the SF-36 (95% CI 0·3–4·8; p=0·028). A treatment effect was not noted for any other outcomes.

**Interpretation:**

To our knowledge, this SPMS cohort is the largest studied to date with comprehensive longitudinal cognitive, neuropsychiatric, and HRQoL assessments. We found evidence of a positive effect of simvastatin on frontal lobe function and a physical quality-of-life measure. Although we found no effect of simvastatin on the other outcome measures, these potential effects warrant confirmation and underline the importance of fully assessing cognition and quality of life in progressive multiple sclerosis treatment trials.

**Funding:**

The Moulton Foundation, the Berkeley Foundation, the Multiple Sclerosis Trials Collaboration, the Rosetrees Trust, a personal contribution from A W Pidgley CBE, and the National Institute for Health Research University College London Hospitals Biomedical Research Centre and University College London.

## Introduction

Cognitive impairment in multiple sclerosis can occur from the earliest stages of the disease and its prevalence can exceed 80% in some studies of secondary progressive multiple sclerosis (SPMS).^1^, ^2^ The cognitive domains most frequently affected in multiple sclerosis are speed of information processing, attention, episodic memory, and executive function.^1^ The effect of cognitive impairment in multiple sclerosis on daily function can be substantial, and greater than the effect of physical disability on quality-of-life measures such as independence, social inclusion, and mood.^3^ In view of this effect, a 2013 international position paper highlighted development of effective interventions to treat cognitive impairment as a key goal in multiple sclerosis.^4^

So far, most studies of cognition in SPMS have been cross-sectional. Longitudinal studies have largely focused on other multiple sclerosis subtypes such as relapsing-remitting multiple sclerosis,^5^ and overall have differed with regard to the rate of progression of cognitive decline. Some studies have reported stability, whereas others have described declines in patient subgroups, in specific domains, or in global cognition.^6^, ^7^, ^8^ However, detailed understanding of the longitudinal pattern of cognitive decline in observational studies, specifically in SPMS, has been limited by small sample sizes, with this group typically investigated as part of larger cohorts that also included patients with different multiple sclerosis subtypes.^8^, ^9^ In a large study of patients with primary progressive multiple sclerosis (PPMS),^7^ baseline impairments of verbal memory, attention, verbal fluency, and spatial reasoning were identified, with cognitive decline occurring in a third of patients after 2 years.

**Research in context**

**Evidence before this study**

We searched MEDLINE (from 1948); Embase (from 1980); and PubMed, the Cochrane Database of Systematic Reviews, the Cochrane Central Register of Controlled Trials, DARE, the Health Technology Assessment database, and the TRIP database (no date restrictions) up to April 30, 2016, for studies with the key words “multiple sclerosis” AND “cognition” OR “neuropsychiatric features” OR “SF-36” OR more general MESH terms for quality of life: “Health Status Indicators”, “Quality of Life”, “Health Status”, “Outcome Assessment (Health Care)”, “Health Surveys”, and “Activities of Daily Living”. We included trials, observational cohort studies (longitudinal and cross-sectional), and systematic reviews. Additionally, we searched abstract books from the European Committee for Treatment and Research in Multiple Sclerosis meetings for the previous 10 years. The search yielded 174 observational cohorts of patients with progressive multiple sclerosis (alone or mixed populations), of which 26 were longitudinal studies of cognition or neuropsychiatric symptoms, or both. After excluding studies in which details of the multiple sclerosis phenotype or results of the cognitive or neuropsychiatric battery were not fully defined, 19 cohorts remained: 11 predominantly examined cognition and included two to 31 patients with secondary progressive multiple sclerosis (SPMS) whose follow-up ranged from 6 months to 17 years. Three Cochrane reviews have been published on pharmacological and neuropsychological treatments of cognition or memory in multiple sclerosis. In the review of pharmacological approaches, seven randomised controlled trials of those assessed by the Cochrane reviewers were suitable for inclusion. Donepezil, ginkgo biloba, memantine, and rivastigmine were examined with trial sizes of 43–126 patients; the maximum number of patients with SPMS was 39, treatment durations were up to 24 weeks, and all trials were judged to be negative. The original review of neuropsychological treatment was updated in 2014 with 20 randomised controlled trials deemed suitable. The individual trial sizes were 15–240 patients, the maximum number of patients with SPMS was 94 (where disease subtype was recorded), and follow-up ranged from 4 weeks to 1 year. There was insufficient evidence for efficacy, in part because of heterogeneity of the evidence base. In 2016, the Cochrane group added seven new trials to its review of techniques for memory rehabilitation, bringing the total number of assessed trials to 15, of which eight were also assessed in the neuropsychological review. Trial sizes were 19–240 patients, the maximum number of patients with SPMS was 94 (where disease subtype was recorded), and the follow-up ranged from 5 weeks to 8 months. Overall, the updated Cochrane review of memory rehabilitation concluded there was limited evidence to support such interventions, particularly with regards to objective memory testing, and that higher-quality studies were needed.

**Added value of this study**

This is, to our knowledge, the largest and longest detailed assessment of cognition and cognitive interventions in SPMS. This study was done in the context of a randomised controlled trial of simvastatin. Baseline assessment showed impairment on tests of frontal lobe function and several memory domains, which is largely consistent with previous findings. After 2 years, verbal and non-verbal memory had declined significantly. Depression worsened, although remained mild. We noted a positive effect from simvastatin on frontal lobe function, although we identified no specific association with frontal atrophy using MRI. We also identified a treatment effect for health-related quality of life (HRQoL—physical component).

**Implications of all the available evidence**

This study reinforces the fact that the domains of memory (verbal and non-verbal) and frontal lobe function (executive function) are preferentially affected in SPMS compared with other cognitive domains and should be targeted in interventional trials. We found a beneficial effect of simvastatin on the frontal cognitive domain, as well as on the physical component of HRQoL, adding to the substantive effect on whole brain atrophy (as well as clinician-reported and patient-reported outcome measures) noted in the original MS-STAT trial. We make the following recommendation for future trial design for cognitive treatments in multiple sclerosis: focus on single phenotypes (eg, SPMS), focus on specific cognitive domains with high baseline impairments (frontal lobe function, episodic memory, and attention and speed of information processing), include a minimum of 12–24 months of follow-up, and develop MRI interim outcomes beyond volumetry, such as other structural measures and functional connectivity models.

Various methods have been used to try to improve cognition in patients with multiple sclerosis, including disease-modifying drugs, acetylcholinesterase inhibitors, cognitive rehabilitation, and exercise, but have yielded variable results with no consistent evidence of benefit.^4^, ^10^ A Cochrane review of pharmacological treatment for memory impairment in patients with multiple sclerosis concluded with no evidence of any useful pharmacological approach (seven trials),^11^ although in another Cochrane review, some support was noted for various neuropsychological rehabilitation techniques (20 trials).^12^ Studies were hampered by mixed disease phenotypes, short durations, and differing outcome measures. A third Cochrane review^13^ concluded that there was limited evidence for memory rehabilition techniques and that more rigorous trial evidence was needed. The effects of exercise on cognition in multiple sclerosis has also been examined, with a pilot study in 42 patients showing some effect of high-intensity aerobic training on learning, memory, and attention.^14^

As well as cognitive impairment, neuropsychiatric symptoms commonly occur in multiple sclerosis, with approximate prevalence of 20% for depression, 9% for anxiety, and 5% for bipolar disorder.^15^ More com-prehensive investigations have identified the occurrence of agitation, irritability, and apathy in multiple sclerosis.^16^ The inter-relationship and directionality between neuropsychiatric symptoms and cognitive impairment remains uncertain, with some reports suggesting an association, but others not.^5^

Compared with the general population, health-related quality of life (HRQoL) is reduced in patients with multiple sclerosis, particularly in those with progressive disease. In a 10-year study using the 36-Item Short Form Survey Instrument (SF-36) to assess HRQoL, decline related predominantly to problems affecting physical status.^17^ In the placebo arm of the IMPACT study^18^ of interferon beta-1a versus placebo in SPMS, the mean change over 2 years in the SF-36 physical component was −0·70 (SD 8·2) and in the psychological component was −1·6 (9·7).

MS-STAT^19^ was a phase 2 trial of high-dose simvastatin (80 mg) in patients with SPMS. A significant 43% reduction in the annualised rate of whole brain atrophy (the primary outcome) was noted, with additional positive effects on clinician-reported and patient-reported outcome measures, namely the Expanded Disability Status Scale (EDSS) and Multiple Sclerosis Impact Scale-29 (MSIS-29v2).

The MS-STAT trial included a pre-planned secondary analysis of cognitive and neuropsychiatric outcome measures together with HRQoL, the results of which are presented here. These secondary analyses were designed to obtain detailed longitudinal imformation on cognitive impairment, neuropsychiatric symptoms and HRQoL, as well as to identify an interventional effect of simvastatin in patients with SPMS.

## Methods

### Study design and patients

MS-STAT was a double-blind, parallel-group, randomised, placebo-controlled trial of simvastatin in patients with SPMS done at three neuroscience centres in the UK between Jan 28, 2008, and Nov 4, 2011. Details of MS-STAT, including the sample size calculation, have been published previously.^19^ In brief, the key study inclusion criteria included age 18–65 years, EDSS score 4·0–6·5, and fulfilment of the revised McDonald diagnostic criteria for multiple sclerosis with evidence of secondary progression over at least the preceding 2 years.^20^ No patients were on disease-modifying treatment.

The protocol was approved by the institutional review board at each study site, and ethics approval was granted by the Berkshire Research Ethics Committee (reference 07/Q1602/73). Patients provided written informed consent before entering the study.

### Randomisation and masking

In MS-STAT, patients were randomly assigned (1:1) to receive simvastatin 80 mg daily or matching placebo for 24 months. Randomisation was by a centralised web-based service with minimisation on the following variables: age (<45 years and ≥45 years), sex (male and female), EDSS (4–5·5 and 6·0–6·5), centre, and assessing physician. Patients, treating physicians, and outcome assessors (including MRI scan analysts) were masked to treatment allocation.

### Procedures

Patients received simvastatin 80 mg daily (two 40 mg tablets inside opaque hard gelatine capsules) or matching placebo (both groups received one tablet per day for the first month before having two per day from then on) for 24 months. Participants were considered compliant with treatment if they reported taking, on average, at least 90% of their drug at a dose of two tablets per day (80 mg simvastatin or matching placebo). Compliance was assessed by the self-reported proportion of capsules taken in the month before assessments at 6, 12, 18, and 24 months. Patients were assessed at baseline and months 1, 6, 12, and 24, with telephone follow-up at months 3 and 18. Cognitive and neuropsychiatric assessments were done at baseline, 12 months, and 24 months, by certified psychologists at three neuroscience centres in southeast England or at patients' homes if needed. HRQoL was assessed using the self-reported SF-36 (version 2), which was completed by patients at baseline, 12 months, and 24 months.

### Outcomes

Analyses of cognitive and neuropsychiatric outcome measures together with HRQoL were prespecified as part of the MS-STAT trial. We used a neuropsychological battery designed specifically to cover a broad range of cognitive domains. The cognitive tests administered (panel) were the National Adult Reading Test (NART; one measure); Wechsler Abbreviated Scale of Intelligence (WASI; seven measures); Graded Naming Test (GNT; one measure); Birt Memory and Information Processing Battery (BMIPB; five measures); cube analysis task from the Visual Object and Space Perception battery (VOSP; one measure); Frontal Assessment Battery (FAB; one measure); and Paced Auditory Serial Addition Test (PASAT-3). The NART (premorbid intelligence quotient [IQ]) and the figure copying measure of the BMIPB (ability to copy figure when placed in front of patient, assessing visuoperceptual function) were done at first visit only as a baseline; thus, 15 longitudinal cognitive outcomes were assessed. Neuropsychiatric status was assessed using the Hamilton Depression Rating Scale (HAM-D) and the Neuropsychiatric Inventory Questionnaire (NPIQ), which has two subscales—severity and distress—representing a brief questionnaire form of the Neuropsychiatric Inventory. HRQoL was assessed using self-reported SF-36 (version 2) and standard scoring methods were used to convert SF-36 (version 2) scores into eight domains of HRQoL from which were derived a physical component summary (PCS), and a mental component summary.

Panel **Prespecified cognitive outcome measures**

• National Adult Reading Test—assessing premorbid IQ*
• Wechsler Abbreviated Scale of Intelligence:
  • Overall IQ—assessing general intellectual function
  • Verbal IQ—assessing verbal IQ
  • Performance IQ—assessing non-verbal IQ
  • Vocabulary—assessing verbal intelligence
  • Similarities—assessing abstract verbal reasoning
  • Block design—assessing spatial perception and visuomotor skills
  • Matrix reasoning—assessing non-verbal abstract reasoning
• Graded Naming Test—assessing semantic memory
• Birt Memory and Information Processing Battery:
  • Story: immediate recall—assessing verbal episodic memory
  • Story: delayed recall—assessing verbal episodic memory
  • Figure copying: ability to copy figure when placed in front of participant*—assessing visuoperceptual functioning
  • Figure copying: immediate recall—assessing non-verbal episodic memory
  • Figure copying: delayed recall—assessing non-verbal episodic memory
• Cube analysis task from the Visual Object and Space Perception battery—assessing spatial perception
• Frontal Assessment Battery—assessing executive function (conceptual thinking, mental flexibility, motor programming, sensitivity to interference, inhibitory control, and environmental autonomy)
• Paced Auditory Serial Addition Test—assessing speed of information processing, attention, and working memory

We also did a post-hoc analysis of frontal lobe volumes (MRI scans were done in the trial at baseline, 12 months, and 25 months, as described previously^19^). The brain was initially parcellated using a multi-atlas propagation and fusion approach, as described by Cardoso and colleagues,^21^ which involves registration of atlas images with associated manual segmentations to each MS-STAT dataset. These propagated segmentations were then fused according to the local similarity between each atlas and the new images. We used the Hammers atlas as the multi-atlas propagation and fusion template database, resulting in 83 non-overlapping brain regions, 24 of which encompassed the frontal lobes. Because these regions were non-overlapping, total frontal lobe volumes were calculated from the summed volumes of these 24 frontal regions. Further details of methods are provided in the appendix (pp 1–2 and 4–7).

### Statistical analysis

All analyses were by intention to treat (ie, included patients in the group to which they were randomly assigned, regardless of compliance with the study protocol). Cognitive scores and HRQoL scores were rescaled with reference to means and SDs of a healthy control group (appendix p 4) to create T scores. On the T score scale, healthy control scores have a mean of 50 and a SD of 10. The reference T scores were calculated using control means and SDs taken from test manuals (NART, WASI, and BMIPB) or reference papers from the published work (WASI and GNT). Age-specific control means were used for the BMIPB, for which age is known to be influential. The VOSP cube analysis and FAB scores are not normally distributed in healthy controls and thus unsuitable for conversion to T scores. Instead, these results are presented as raw scores, as are the measures of neuropsychiatric status and PASAT-3 scores. PASAT-3 scores were normally distributed, but have been previously reported as raw scores and are retained in this format in this Article to preserve consistency with the previous report.^19^ Impairment on cognitive scores and HRQoL scores at baseline was defined as a score of more than 1·5 SDs below the mean of the reference healthy control group (appendix p 4). On the T score scale this is a score less than 35. Neuropsychiatric symptoms were assessed in terms of prevalence and severity. Since these are symptoms indicative of disease and thus not normally present in healthy control individuals, severity was not investigated in terms of differences from normative values.

We used linear mixed models to examine how HRQoL, cognitive scores, neuropsychiatric scores, and frontal lobe volumes changed between baseline, 12 months, and 24 months, and to assess the difference in means between the placebo and simvastatin groups at 12 months and 24 months. For analysis of cognitive and neuropsychiatric scores, we used a mixed effect model to compare the mean of each outcome between the placebo and simvastatin groups at the visits at 12 months and 24 months, with adjustment for minimisation variables. In the model, measurements made at baseline, 12 months, and 24 months were classed as three correlated outcomes. Interactions between treatment group and visit were included to estimate the treatment effects, with that at baseline set as zero to reflect the fact that randomisation ensures that the true treatment effect at baseline is zero. We adjusted for minimisation variables by including interactions between visit and each variable. An unstructured residual variance–covariance matrix allowed for assessment of the correlation between repeated measures in the same patient. Data were included from all patients who had an outcome measured at one or more of the visits, providing an unbiased estimate of the treatment effect under the assumption that the model is correctly specified, and that data are missing at random. For each outcome, we estimated three separate fitted mean changes between baseline and 24 months from the relevant mixed effect model. First, we calculated the mean change for the cohort overall for a population with the same distribution of baseline values of the minimisation variables as the cohort as a whole, and 1:1 randomisation to treatment group. The other two mean changes were those for populations with the same distribution of minimisation variables as above, but with (1) all patients randomly assigned to placebo, and (2) all patients randomly assigned to simvastatin.

The mean rates of frontal lobe atrophy were compared between the two treatment groups using a linear mixed model for change per year. The model included an interaction between treatment group and time since baseline MRI as well as minimisation variables, MRI site, and their interactions with time. The treatment effect was set as zero at baseline, as described earlier. The model included a random intercept and random slope as correlated random effects, to allow for repeated measures.

The assumption that residuals follow a homoscedastic normal distribution, which is required for valid parametric statistical inference, was not met for VOSP, FAB, NPIQ distress and NPIQ severity, or HRQoL domains of physical functioning, role limitations–physical, or role limitations–emotional. For these variables, inference was based on non-parametric, bias-corrected and accelerated bootstrap 95% CIs calculated from 2000 replications stratified by treatment group and clustered by patient. As a result of bootstrapping, p values cannot be provided. However, statistical significance (p<0·05) can be inferred by whether or not the 95% CI crosses zero. Since we class all the variables as of independent scientific interest, no formal statistical adjustments for multiple comparisons were made. However, the data must be interpreted with caution in view of the number of variables analysed. We used Stata (version 14.1) for all analyses. MS-STAT is registered with ClinicalTrials.gov, number NCT00647348.

### Role of the funding source

The funders of the study had no role in study design, data collection, data analysis, data interpretation, writing of the report, or the decision to submit the manuscript. CF, JMN, JC, and RN had full access to all the data in the study; DC, SB, and DW had access to the cognitive data; and MJC and SO had access to the frontal lobe volumetry data. The corresponding author had final responsibility for the decision to submit for publication.

## Results

**Figure 1:**
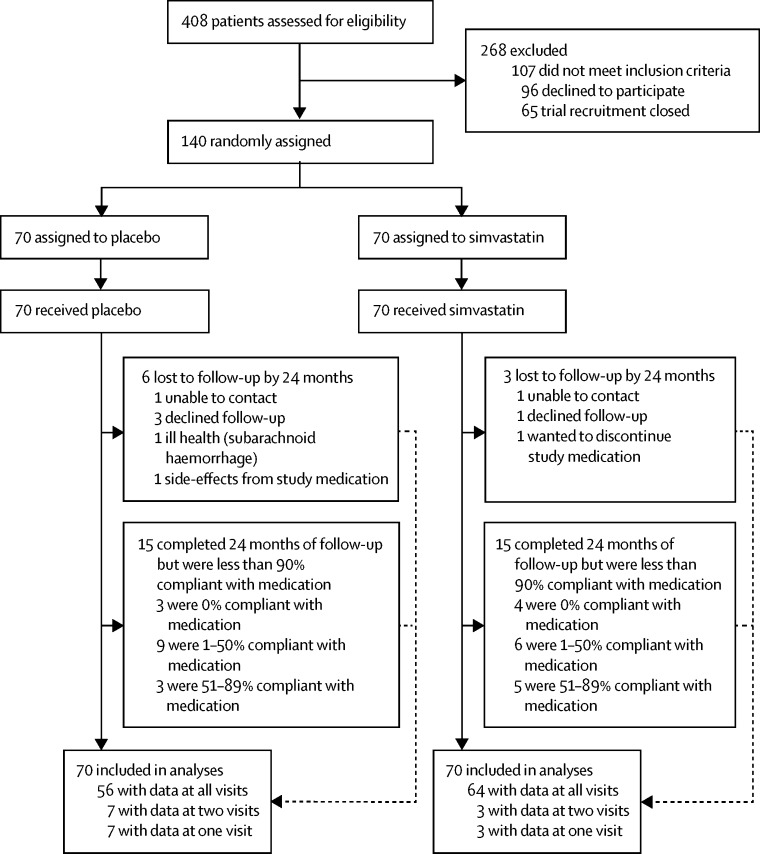
Trial profile

140 patients were recruited to the MS-STAT trial (figure 1). Patient demographics were similar between groups, with a mean age of 51·3 years (SD 6·9), multiple sclerosis duration of 21·2 years (8·6), and SPMS duration of 7·2 years (5·2; table 1). T scores of around 50 suggested that the premorbid IQ (derived from the NART) was in the normal range (table 1). At baseline, both study groups had similar scores on cognitive, neuropsychiatric, and HRQoL measures.

**Table 1:** Baseline characteristics of participants

			**Combined**		**Placebo**		**Simvastatin**	
			N	Mean (SD)	N	Mean (SD)	N	Mean (SD)
Age (years)			140	51·3 (6·9)	70	51·1 (6·8)	70	51·5 (7·0)
Multiple sclerosis duration (years)			140	21·2 (8·6)	70	20·3 (8·8)	70	22·1 (8·3)
SPMS duration (years)			140	7·2 (5·2)	70	7·1 (4·8)	70	7·3 (5·6)
Expanded Disability Status Scale			140	5·8 (0·8)	70	5·9 (0·8)	70	5·8 (0·8)
Education (years)			132	13·5 (3·2)	66	13·4 (3·3)	66	13·7 (3·1)
Premorbid verbal IQ (NART)*			135	52·7 (6·5)	68	51·7 (6·8)	67	53·7 (6·1)
Premorbid performance IQ (NART)*			135	54·7 (4·6)	68	54·0 (4·8)	67	55·4 (4·4)
Premorbid full-scale IQ(NART)*			135	53·9 (6·2)	68	53·0 (6·5)	67	54·9 (5·9)
Cognitive scores								
	Wechsler Abbreviated Scale of Intelligence*							
		Overall IQ	130	53·1 (9·6)	66	53·4 (9·6)	64	52·8 (9·8)
		Verbal IQ	131	54·5 (9·5)	66	54·1 (9·7)	65	54·8 (9·3)
		Performance IQ	130	51·1 (9·5)	66	51·8 (9·1)	64	50·4 (10·0)
		Vocabulary	130	56·0 (10·2)	65	55·9 (9·3)	65	56·1 (11·1)
		Similarities	129	51·4 (9·3)	63	51·0 (9·6)	66	51·8 (9·0)
		Block design	129	48·2 (9·2)	64	48·6 (9·0)	65	47·7 (9·5)
		Matrix reasoning	129	53·4 (10·5)	65	54·6 (9·4)	64	52·2 (11·4)
	Graded Naming Test*		130	53·2 (12·2)	66	52·8 (12·0)	64	53·6 (12·6)
	Birt Memory and Information Processing Battery*							
		Story immediate	131	43·2 (10·7)	66	42·7 (9·8)	65	43·7 (11·7)
		Story delay	131	43·7 (10·7)	66	43·1 (9·4)	65	44·3 (11·9)
		Figure copying	130	48·5 (11·5)	66	49·3 (12·1)	64	47·7 (10·8)
		Figure copying: immediate recall	130	41·0 (11·1)	66	40·5 (11·2)	64	41·5 (11·0)
		Figure copying: delayed recall	130	46·4 (9·2)	66	46·4 (9·4)	64	46·4 (9·1)
	VOSP cube analysis		132	9·2 (1·6)	67	9·3 (1·5)	65	9·1 (1·7)
	Frontal Assessment Battery		133	16·1 (2·4)	67	15·9 (2·5)	66	16·3 (2·4)
	Paced Auditory Serial Addition Test		134	34·2 (15·0)	67	33·7 (16·1)	67	34·8 (13·8)
Neuropsychiatric scores								
	Hamilton Depression Rating Scale		133	9·2 (6·2)	67	9·3 (6·1)	66	9·1 (6·3)
	NPIQ severity		112	4·5 (4·7)	55	4·9 (5·2)	57	4·1 (4·1)
	NPIQ distress		112	5·0 (5·5)	55	5·6 (6·1)	57	4·3 (4·7)
Health-related quality of life*								
	Mental component score		122	48·9 (11·1)	59	47·9 (12·6)	63	49·8 (9·5)
	Physical component score		122	33·7 (8·7)	59	32·8 (8·5)	63	34·5 (8·9)
	Physical functioning		130	27·1 (8·8)	62	25·6 (7·9)	68	28·4 (9·3)
	Role limitations physical		130	35·8 (9·9)	64	36·0 (10·2)	66	35·7 (9·7)
	Bodily pain		135	48·4 (11·4)	66	46·3 (11·6)	69	50·4 (10·9)
	General health		131	39·6 (10·7)	65	39·1 (9·7)	66	40·1 (11·6)
	Vitality		130	42·4 (9·0)	65	42·2 (9·5)	65	42·7 (8·4)
	Social functioning		134	39·2 (11·8)	66	38·0 (11·9)	68	40·3 (11·7)
	Role limitations emotional		133	44·3 (13·7)	66	43·8 (14·0)	67	44·8 (13·5)
	Mental health		131	47·3 (9·9)	64	45·9 (10·6)	67	48·6 (9·1)

Premorbid IQ data were considered a baseline demographic characteristic, against which cognitive data could be compared. SPMS=secondary progressive multiple sclerosis. IQ=intelligence quotient. NART=National Adult Reading Test. VOSP=Visual Object and Space Perception. NPIQ=Neuropsychiatric Inventory Questionnaire.

* T score (mean 50 [SD 10] in healthy reference population).

**Figure 2:**
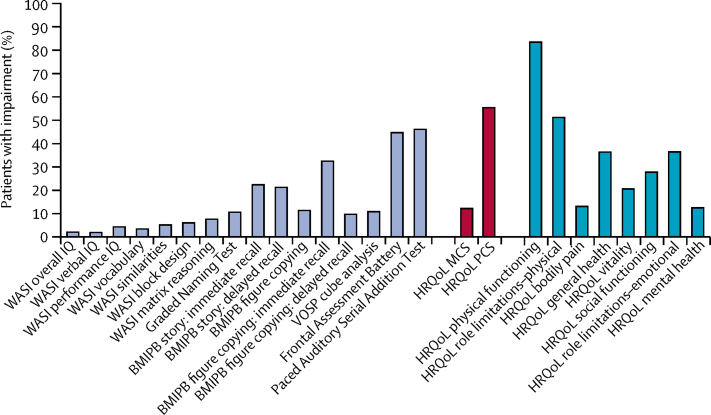
Percentage of patients impaired in each measure at baseline in both groups combined Numerators and denominators used to calculate these percentages are provided in the appendix (p 8). WASI=Wechsler Abbreviated Scale of Intelligence. IQ=intelligence quotient. BMIPB=Birt Memory and Information Processing Battery. VOSP=Visual Object and Space Perception. HRQoL=health-related quality of life. MCS=mental component score. PCS=physical component score.

The proportion of patients with cognitive impairment at baseline differed across cognitive domains (figure 2; appendix p 8). Three (2%) of 131 to ten (8%) of 129 patients showed impairment at baseline of general intellectual functioning in verbal, visuomotor, or abstract reasoning domains, as measured by the WASI. 14 (11%) of 130 were impaired in semantic memory (GNT). Between 13 (10%) of 130 and 43 (33%) of 130 patients were impaired on tests of immediate and delayed verbal and non-verbal episodic memory recall (BMIPB story and figure scores), with 15 (11%) of 132 impaired on higher visual processing using the VOSP cube analysis. 60 (45%) of 133 patients exhibited impairment in executive function assessed by the FAB, and 62 (46%) of 134 were impaired on the PASAT-3.

HAM-D scores suggested that 76 (57%) of 133 patients had depression: 47 (35%) mild, 21 (16%) moderate, and eight (6%) severe or very severe. Although the NPIQ does not subdivide neuropsychiatric symptoms into mild, moderate, or severe categories, NPIQ severity (degree of symptoms in the patient) and distress (effect on caregiver) scores of 4·5 (SD 4·7) and 5·0 (5·5) were above mean scores of 1·5 and 1·6 for cognitively intact older adults, but below the mean scores of 7·9 and 9·4 associated with dementia disorders (appendix p1, p4).

For HRQoL, 15 (12%) of 122 patients had impairment in the MCS and 68 (56%) in the PCS at baseline (figure 2; appendix p 8). Of the eight HRQoL domains, the highest proportion with impairment was for physical functioning (109 [84%] of 130), followed by role limitations–physical (67 [52%]). 18 (13%) of 135 patients had impairment on bodily pain and 17 (13%) of 135 had impairment on mental health.

At 24 months, frontal lobe function (FAB scores) was significantly better with simvastatin treatment than with placebo (difference 1·2 points, 95% CI 0·2 to 2·3; table 2). FAB score increased from baseline in the simvastatin group (change 0·3 points, 95% CI −0·4 to 0·9), whereas it decreased in the placebo group (change −0·9, −1·9 to −0·1; table 2). There was no significant difference between the placebo and simvastatin groups for any other cognitive or neuropsychiatric outcome (table 2), but we noted weak evidence of improvement in the simvastatin group compared with placebo at 24 months in the WASI block T score (difference 2·1, 95% CI −0·1 to 4·3; p=0·064) and PASAT-3 score (3·9 points, −0·3 to 8·1; p=0·070). The appendix (p 9) shows the individual patient changes in the FAB score.

**Table 2:** Changes in cognitive and neuropsychiatric scores, health-related quality of life, and frontal lobe volume between baseline and 24 months

		**Combined**			**Placebo**		**Simvastatin**		**Treatment effect (95% CI)**	**p value**
		N	Change (95% CI)	p value	N	Change (95% CI)	N	Change (95% CI)		
**Cognitive scores**										
Wechsler Abbreviated Scale of Intelligence*										
	Overall IQ	135	−0·2 (−1·4 to 0·9)	0·67	68	−0·3 (−1·9 to 1·3)	67	−0·2 (−1·7 to 1·3)	0·1 (−2·0 to 2·2)	0·92
	Verbal IQ	136	−1·4 (−2·8 to 0·0)	0·058	68	−1·2 (−3·2 to 0·7)	68	−1·5 (−3·4 to 0·3)	−0·3 (−2·8 to 2·2)	0·82
	Performance IQ	135	0·8 (−0·2 to 1·8)	0·13	68	0·3 (−1·1 to 1·8)	67	1·2 (−0·1 to 2·6)	0·9 (−1·0 to 2·9)	0·35
	Vocabulary	137	−1·2 (−3·0 to 0·5)	0·17	68	−1·3 (−3·7 to 1·1)	69	−1·1 (−3·4 to 1·2)	0·2 (−2·9 to 3·3)	0·90
	Similarities	136	−0·8 (−2·3 to 0·7)	0·30	67	−0·6 (−2·7 to 1·5)	69	−1·0 (−2·9 to 1·0)	−0·3 (−3·1 to 2·4)	0·80
	Block design	136	0·6 (−0·5 to 1·7)	0·29	68	−0·4 (−2·0 to 1·2)	68	1·6 (0·1 to 3·2)	2·1 (−0·1 to 4·3)	0·064
	Matrix reasoning	136	0·9 (−0·8 to 2·6)	0·30	68	1·4 (−1·0 to 3·8)	68	0·4 (−1·9 to 2·7)	−1·0 (−4·2 to 2·2)	0·55
Graded Naming Test*		135	−0·5 (−1·7 to 0·7)	0·44	68	−0·3 (−2·0 to 1·4)	67	−0·7 (−2·3 to 0·9)	−0·4 (−2·7 to 1·9)	0·75
Birt Memory and Information Processing Battery*										
	Story: immediate recall	136	−2·5 (−4·5 to −0·4)	0·018	68	−3·0 (−6·0 to −0·1)	68	−2·0 (−4·7 to 0·8)	1·1 (−2·9 to 5·0)	0·60
	Story: delayed recall	136	−5·7 (−7·8 to −3·6)	<0·0001	68	−6·5 (−9·5 to −3·4)	68	−5·0 (−7·8 to −2·1)	1·5 (−2·6 to 5·7)	0·47
	Figure copying: immediate recall	135	−1·8 (−4·1 to 0·5)	0·12	68	−1·4 (−4·8 to 1·9)	67	−2·2 (−5·3 to 0·8)	−0·8 (−5·2 to 3·6)	0·72
	Figure copying: delayed recall	135	−6·8 (−8·7 to −4·8)	<0·0001	68	−7·0 (−9·9 to −4·2)	67	−6·5 (−9·1 to −3·9)	0·5 (−3·4 to 4·4)	0·79
VOSP cube analysis†		137	0·0 (−0·3 to 0·3)	..	69	0·0 (−0·4 to 0·3)	68	0·0 (−0·4 to 0·4)	0·0 (−0·5 to 0·5)	..
Frontal Assessment Battery†		138	−0·3 (−0·9 to 0·3)	..	69	−0·9 (−1·9 to −0·1)	69	0·3 (−0·4 to 0·9)	1·2 (0·2 to 2·3)	..
Paced Auditory Serial Addition Test		140	2·0 (−0·2 to 4·2)	0·074	70	0·0 (−3·1 to 3·2)	70	3·9 (1·0 to 6·8)	3·9 (−0·3 to 8·1)	0·070
**Neuropsychiatric measures**										
Hamilton Depression Rating Scale		138	2·8 (1·5 to 4·0)	<0·0001	69	3·3 (1·6 to 5·0)	69	2·3 (0·7 to 3·8)	−1·0 (−3·2 to 1·2)	0·37
NPIQ severity†		127	0·5 (−0·7 to 1·7)	..	64	0·7 (−0·7 to 2·6)	63	0·2 (−1·4 to 1·6)	−0·6 (−2·7 to 1·6)	..
NPIQ distress†		127	1·0 (−0·4 to 2·5)	..	64	1·6 (−0·5 to 3·9)	63	0·3 (−1·3 to 1·9)	−1·3 (−3·9 to 1·1)	..
Health-related quality of life*										
Mental component score		136	−0·8 (−3·0 to 1·4)	0·48	67	−0·7 (−3·8 to 2·3)	69	−0·8 (−3·7 to 2·0)	−0·1 (−4·0 to 3·8)	0·96
Physical component score		136	−0·6 (−1·9 to 0·7)	0·36	67	−1·9 (−3·6 to −0·1)	69	0·7 (−1·0 to 2·3)	2·5 (0·3 to 4·8)	0·028
Physical functioning†		138	−2·5 (−3·8 to −1·4)	..	68	−2·3 (−4·0 to −0·7)	70	−2·8 (−4·4 to −1·4)	−0·5 (−2·8 to 1·5)	..
Role limitations–physical†		137	0·8 (−1·2 to 2·7)	..	68	−0·5 (−2·9 to 1·9)	69	2·1 (−0·6 to 4·8)	2·6 (−0·7 to 6·0)	..
Bodily pain		139	−1·0 (−2·6 to 0·7)	0·24	69	−1·7 (−4·0 to 0·7)	70	−0·3 (−2·5 to 1·9)	1·4 (−1·7 to 4·4)	0·39
General health		139	−0·1 (−1·6 to 1·4)	0·89	69	0·1 (−2·1 to 2·3)	70	−0·3 (−2·3 to 1·7)	−0·4 (−3·3 to 2·4)	0·77
Vitality		137	0·2 (−1·4 to 1·8)	0·82	68	0·3 (−1·9 to 2·6)	69	0·0 (−2·1 to 2·2)	−0·3 (−3·2 to 2·7)	0·85
Social functioning		139	−1·9 (−4·2 to 0·5)	0·12	69	−0·8 (−4·0 to 2·4)	70	−2·9 (−5·9 to 0·1)	−2·1 (−6·2 to 2·0)	0·31
Role limitations–emotional†		139	−1·4 (−4·1 to 1·3)	..	69	−2·3 (−6·0 to 1·3)	70	−0·5 (−4·2 to 2·9)	1·8 (−2·9 to 6·3)	..
Mental health		138	−1·3 (−3·2 to 0·6)	0·19	68	−0·5 (−3·1 to 2·1)	70	−2·0 (−4·5 to 0·4)	−1·5 (−4·9 to 1·9)	0·38
**MRI**										
Frontal lobe atrophy (mL/year)‡		140	−1·0 (−1·3 to −0·6)	<0·0001	70	−1·0 (−1·5 to −0·4)	70	−0·9 (−1·4 to −0·4)	0·0 (−0·7 to 0·7)	0·97

Imaging was done between baseline and 25 months. IQ=intelligence quotient. VOSP=Visual Object and Space Perception. NPIQ=Neuropsychiatric Inventory Questionnaire.

* T score.

† As a result of bootstrapping, p values cannot be provided; however, significance (p<0·05) can be inferred by the 95% CI not crossing zero.

‡ Post-hoc analysis.

In terms of HRQoL, there was a significant treatment effect on PCS (difference 2·5 points, 95% CI 0·3 to 4·8; p=0·028), which corresponded to a mean increase in PCS of 0·7 (95% CI −1·0 to 2·3) in the simvastatin group and a decrease of 1·9 (0·1 to 3·6) in the placebo group. The appendix (pp 9, 10) shows the individual patient changes in PCS and MCS. There was no evidence of differences between the placebo and simvastatin groups for MCS or any of the eight individual HRQoL domains (table 2).

**Figure 3:**
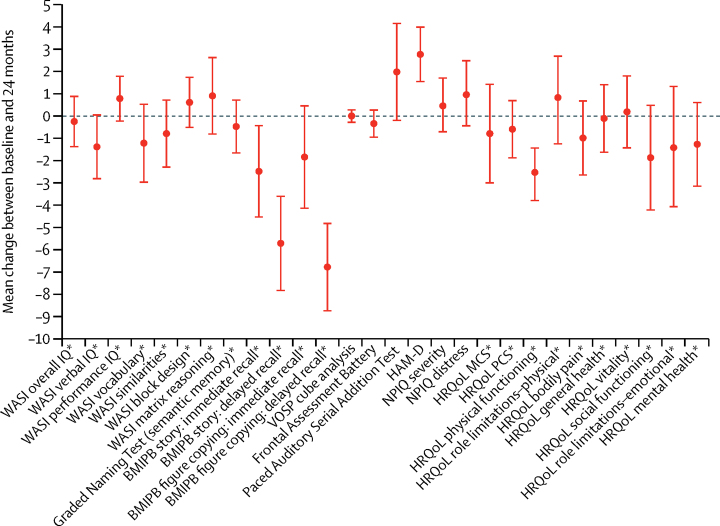
Change in cognitive scores, neuropsychiatric scores, and health-related quality of life between baseline and 24 months in both groups combined Bars are 95% CIs. In all tests, except HAM-D and NPIQ, a positive change suggests improvement. WASI=Wechsler Abbreviated Scale of Intelligence. IQ=intelligence quotient. BMIPB=Birt Memory and Information Processing Battery. VOSP=Visual Object and Space Perception. HAM-D=Hamilton Depression Rating Scale. NPIQ=Neuropsychiatric Inventory Questionnaire. HRQoL=health-related quality of life. MCS=mental component score. PCS=physical component score. *T score.

Figure 3 shows mean changes in cognitive, neuropsychiatric, and HRQoL outcomes between baseline and 24 months. General intellectual functioning as measured by WASI did not change significantly across the duration of the study. There was no significant change in naming and verbal semantic memory (GNT). For the cohort as a whole, we noted the greatest decline on measures of delayed episodic memory recall (BMIPB), with the mean T-score worsening by 5·7 points (95% CI −7·8 to −3·6; p<0·0001) for delayed verbal recall and by 6·8 points (–8·7 to −4·8; p<0·0001) for non-verbal recall. Immediate verbal episodic recall showed a mean decline of 2·5 points (–4·5 to −0·4; p=0·018), but the 1·8 point drop in immediate non-verbal episodic recall was not significant (–4·1 to 0·5; p=0·12). There was no significant change in spatial perception (VOSP). There was an increase of 2·8 points (1·5 to 4·0) on the HAM-D (p<0·0001), although 64 (68%) of 94 patients' scores at 24 months still suggested either no depression or mild depression. There was no significant change in mean NPIQ severity or distress scores. Neither the MCS nor PCS HRQoL changed significantly between baseline and 24 months for the cohort as a whole. However, as mentioned earlier, the mean PCS decreased in the placebo group, but did not change substantially in the simvastatin group. Of the individual HRQoL domains, there was a 2·5 point decline in mean physical functioning score, whereas the other domains did not change significantly between baseline and 24 months.

In view of the effect on frontal lobe function, we did a post-hoc analysis using frontal lobe volumetry, which was judged to be the most appropriate technique for our stated region of interest. We noted no significant difference in the rate of change in frontal lobe volume between the simvastatin and placebo groups (table 2). There was no evidence of an association between change in FAB score between baseline and 24 months and change in frontal lobe volume between baseline and 25 months, either in the placebo (Spearman rank correlation 0·17; p=0·29; n=40) or simvastatin (–0·19; p=0·21; n=45) group.

## Discussion

This is, to our knowledge, the largest reported cohort of patients with SPMS to have undergone detailed longitudinal assessment of cognition, neuropsychiatric status, and HRQoL. At baseline, the most prominent cognitive deficits were in attention and speed of information processing, frontal lobe function, verbal and non-verbal recall, and working memory. This profile is similar to that reported previously in other cross-sectional SPMS groups.^2^, ^22^, ^23^, ^24^

In terms of the effect of simvastatin on cognition, over the 24-month trial, changes in the overall cognitive profile were similar for both simvastatin and placebo groups, with no effect of simvastatin on most cognitive domains. However, there was evidence of a positive treatment effect on frontal lobe function, assessed using the FAB. Overall cognitive decline was greatest in verbal and non-verbal episodic memory recall, without significant changes in general intellectual function, naming, or higher visual processing. Although the much smaller cohorts of previous longitudinal SPMS studies (generally 20% of this study) precludes like-for-like comparison, changes over time were noted in episodic memory, learning, attention, speed of information, and visual processing.^6^, ^8^, ^9^

In terms of neuropsychiatric outcome measures, over 24 months, depression increased significantly, as determined by the HAM-D score, and there was a non-significant increase in NPIQ severity and distress scores, although no treatment effect was noted on any neuropsychiatric outcome measure.

The potential treatment effect on frontal lobe function warrants further discussion. Several studies have described impaired frontal lobe function in multiple sclerosis,^22^, ^23^, ^24^, ^25^, ^26^ and a recent, large cross-sectional study,^25^ including 74 patients with SPMS, revealed a heavy burden of executive dysfunction in all progressive multiple sclerosis subtypes. Although Ruano and colleagues^25^ showed patients with PPMS more frequently had executive dysfunction compared with patients with other subtypes of multiple sclerosis, others have shown frontal lobe capabilities to be affected to a greater extent in SPMS than in PPMS.^22^, ^23^, ^24^ In one study of patients with relapsing-remitting multiple sclerosis, impairment of frontal executive function was noted in the context of otherwise intact cognitive function,^26^ suggesting that this cognitive domain might be one of the earliest affected in multiple sclerosis. However, the reason for the apparently selective effect of simvastatin on frontal lobe function is unclear. There is no obvious pharmacological reason based on the current understanding of the mode of action of simvastatin that would result in a preferential improvement in frontal lobe function. Therefore, this finding might be due in part to the study population, in particular the level of impairment at baseline, with the FAB being one of the tests in which the greatest proportion of patients were affected (about 45%). As such, any treatment effect on other cognitive tests might have been more subtle, because proportionally fewer patients were impaired, and this study did not have sufficient power to detect these effects.

Various methods have been used in previous studies to assess frontal lobe function in multiple sclerosis, including bespoke frontal lobe test batteries,^24^ or as part of tests within global batteries such as the MindStreams Global Assessment Battery^23^ or Brief Repeatable Battery,^7^, ^9^ in some cases augmented by additional frontal lobe assessments.^25^ The FAB was chosen for this study in view of its ability to probe differing aspects of frontal lobe function, for which it has been used widely in the study of patients with frontotemporal dementia and other neurodegenerative disorders affecting the frontal lobes. Furthermore, the reproducibility and ease of administration of the FAB confers advantages in terms of application to large multiple sclerosis cohorts. The FAB has previously been used in patients with multiple sclerosis, principally in studies focused on assessment of quality of life and coping strategies, but also as part of an executive function battery.^2^, ^27^ To our knowledge, this is the first study to use the FAB as an independently reported cognitive outcome measure within a longitudinal interventional study, and the study findings show the importance of including a comprehensive assessment of frontal lobe function in future multiple sclerosis interventional studies, either as an individual outcome measure or in addition to current batteries such as the Brief International Cognitive Assessment for MS.^5^

By contrast with the treatment effect on whole brain atrophy rates reported previously,^19^ we noted no significant effect on rates of frontal lobe atrophy, and there was no significant correlation between FAB scores and rates of frontal lobe atrophy. Several potential explanations exist for this apparent dissociation. Other imaging measures might be superior predictors of executive function than atrophy.^5^, ^28^ Corpus callosum atrophy might outperform other imaging markers of cognitive dysfunction, such as grey or white matter fraction, and tracked over 17 years, was associated with cognitive dysfunction in multiple sclerosis subtypes including SPMS;^29^ other studies have used resting-state functional MRI to identify an association between cognitive impairment and altered functional connectivity.^5^ More recently, techniques such as thalamic volume and activation^28^, ^30^ and cortical lesions visible via high-field MRI^31^ have shown associations with cognition.

We also showed evidence that simvastatin treatment had a positive effect on physical HRQoL, as measured using the SF-36 (version 2) PCS. This finding is consistent with the positive treatment effect on the EDSS and MSIS-29v2 (especially the physical subscale) previously reported.^19^ The absence of effect on the mental component scale of the HRQoL is consistent with the absence of effect on the neuropsychiatric outcome measures and MSIS-29v2 psychological subscale.

This study has several limitations. First, in view of the need for comprehensive assessment of cognition and neuropsychiatric status, this study involved analysis of data from 15 cognitive outcomes and three neuropsychiatric outcomes. Caution is therefore warranted in the interpretation of the evidence of a positive treatment effect on the measure of frontal lobe function and confirmation of this finding in independent studies is needed. The fluctuating nature of disease activity in all patients with multiple sclerosis, and its susceptibility to environmental factors such as heat and concomitant illness, are confounders in the study of cognitive function in multiple sclerosis.^32^ Such fluctuations might lead to variability in test performance, which would reduce the power of the study to detect an effect of treatment on cognition. Although patients were screened for possible concurrent acute medical disorders at each study visit, the possibility of variability in test performance as a result of external factors cannot be entirely excluded. The effect of patient dropout on data analysis also needs to be considered. However, this effect was offset in this study by the use of a statistical model that maximised data inclusion by incorporating all available cognitive assessments for all patients, thus ensuring that the estimated treatment effect was unbiased if data were missing at random.

In conclusion, we found evidence of a positive effect of treatment with high-dose simvastatin on frontal lobe function and a physical quality-of-life measure, adding to our previous findings of a treatment effect on the annualised rate of whole brain atrophy.^19^

## Footnotes

* Assessed at baseline only; therefore, 15 cognitive outcomes were reported longitudinally. IQ=intelligence quotient.
