# Supplementary material for: Effect of high-dose simvastatin on cognitive, neuropsychiatric, and health-related quality-of-life measures in secondary progressive multiple sclerosis: secondary analyses from the MS-STAT randomised, placebo-controlled trial
Source: Lancet Neurol. 2017 Aug;16(8):591–600. doi: 10.1016/S1474-4422(17)30113-8 (PMC5507768; doi:10.1016/S1474-4422(17)30113-8)
Supplement: Supplementary appendix [file mmc1.pdf]

THE LANCET
Neurology

Supplementary appendix

This appendix formed part of the original submission and has been peer reviewed. We post it as supplied by the authors.

Supplement to: Chan C, Binks S, Nicholas JM, et al. Effect of high-dose simvastatin on cognitive, neuropsychiatric, and health-related quality-of-life measures in secondary progressive multiple sclerosis: secondary analyses from the MS-STAT randomised, placebo-controlled trial. *Lancet Neurol* 2017; published online June 6. http://dx.doi.org/10.1016/S1474-4422(17)30113-8.

Web appendix 1: References for methods for neuropsychological testing, frontal lobe volumetric and statistical analysis

Carpenter JR, Kenward MG. Missing data in randomised controlled trials – a practical guide. Birmingham: National Institute for Health Research; 2008.

Coughlan AK, Oddy MJ, Crawford JR. BIRT Memory and Information Processing Battery (BMIPB). Horsham, England: Brain Injury Rehabilitation Trust; 2007.

Cummings JL, Mega M, Gray K, Rosenberg-Thompson S, Carusi DA, Gornbein J. The neuropsychiatric inventory: comprehensive assessment of psychopathology in dementia. Neurology. 1994 Dec; 44 (12): 2308-14.

Dubois B, Slachevsky A, Litvan I, Pilon B. The FAB. A frontal assessment battery at bedside. Neurology. 2000 Dec 12; 55 (11): 1621-6.

Gronwall DM. Paced auditory serial-addition task: a measure of recovery from concussion. 1977 Apr; 44 (2): 367-73.

Hamilton M. A rating scale for depression. J Neurol Neurosurg Psychiatry. 1960 Feb; 23: 56-62.

Hammers A, Allom R, Koepp MJ *et al*. Three-dimensional maximum probability atlas of the human brain, with particular reference to the temporal lobe. Hum Brain Mapp. 2003 Aug; 19 (4): 224-47.

Kaufer DI, Cummings JL, Ketchel P *et al*. Validation of the NPI-Q, a brief clinical form of the Neuropsychiatric Inventory. J Neuropsychiatry Clin Neurosci. 2000 Spring; 12 (2): 233-9.

McKenna P, Warrington EK. The graded naming test. Windsor, England: NFER-Nelson; 1983.

Nelson HE. The National Adult Reading Test (NART): test manual. 2nd ed. Windsor: NFER-Nelson; 1991.

Ryan JR, Carruthers CA, Miller LJ et al. Exploratory Factor Analysis of the Wechsler Abbreviated Scale of Intelligence (WASI) in Adult Standardization and Clinical Samples. Appl Neuropsychol. 2003;10 (4):252-6

Saris-Baglama, RN, Dewey, CJ, Chisholm GB *et al*. QualityMetric Health Outcomes™ Scoring Software. 4.0 ed. Lincoln, USA: QualityMetric Incorporated; 2010.

Verbeke G, Molenberghs G. Linear mixed models for longitudinal data. New York: Springer-Verlag; 2000.

Ware JE Jr, Sherbourne CD. The MOS 36-item short-form health survey (SF-36). I. Conceptual framework and item selection. Med Care. 1992 Jun; 30 (6): 473-83.

Warrington EK, James M. The Visual Object and Space Perception Battery (VOSP). Bury St. Edmunds, England: Thames Valley Test Co; 1991. (Now available through Pearson Assessment: info@pearsonclinical.co.uk).

Warrington EK. The graded naming test: a restandardisation. Neuropsychological Rehabilitation: an International Journal. 1997 Apr; 7 (2): 143–146.

Wechsler D. Wechsler Abbreviated Scale of Intelligence (WASI). San Antonio, TX: Psychological Corporation; 1999.

Zimmerman M, Martinez JH, Young D, Chelminski I, Dalrymple K. Severity classification on the Hamilton Depression Rating Scale. J Affect Disord. 2013 Sep 5; 150 (2): 384-8.

Web-appendix 2: List of abbreviations

BICAMS - Brief International Cognitive Assessment for MS

BMIPB - Birt Memory and Information Processing Battery

EDSS – Expanded Disability Status Scale

FAB - Frontal Assessment Battery

GNT – Graded Naming Test

HAM-D – Hamilton Depression Scale

HRQL – health related quality of life

MAPF - multi-atlas propagation and fusion

MCS – Mental Component Summary (of SF-36)

MSIS-29 – Multiple Sclerosis Impact Scale

NART – National Adult Reading Test

NPIQ - Neuropsychiatric Inventory Questionnaire

PASAT – Paced Auditory Serial Addition Test

PCS – Physical Component Summary (of SF-36)

SF-36 – 36-Item Short Form- Survey

VOSP - Visual Object and Space Perception

WASI - Wechsler Abbreviated Scale of Intelligence

Web-appendix 3: Healthy control data sources for the cognitive battery

The data for T-scores were taken from the instruction manuals or literature (as stated under methods) for NART, WASI, GNT and BMIPB.

Further information on cohorts who participated for standardisation of these tests is:

- NART – 120 patients aged 20-70 attending the National Hospital for Nervous Diseases (Nelson HE. The National Adult Reading Test (NART): test manual. 2nd ed. Windsor: NFER-Nelson; 1991.)
- WASI –1145 healthy US citizens aged 17-89 (Ryan JR, Carruthers CA, Miller LJ et al. Exploratory Factor Analysis of the Wechsler Abbreviated Scale of Intelligence (WASI) in Adult Standardization and Clinical Samples. Appl Neuropsychol. 2003;10(4):252-6.)
- GNT – 305 normal controls aged 18-77 attending the Dept of Psychology at the NHNN, aged 18-77 (mean 40.6) (Warrington EK. The graded naming test: a restandardisation. Neuropsychological Rehabilitation: an International Journal. 1997 Apr; 7 (2): 143–146.)
- BMIPB – 300 participants from the UK general population age range 16-89, reflecting as closely as possible demographics of the UK general population (Coughlan AK, Oddy MJ, Crawford JR. BIRT Memory and Information Processing Battery (BMIPB). Horsham, England: Brain Injury Rehabilitation Trust; 2007.)

For the NPIQ, healthy control values (32 cognitively intact elderly controls) were taken from:

- Leonard M, McInerney S, McFarland J et al. Comparison of cognitive and neuropsychiatric profiles in hospitalised elderly medical patients with delirium, dementia and comorbid delirium-dementia. BMJ Open. 2016 Mar 8; 6 (3): e009212.

Web-appendix 4: MRI acquisition parameters

MRI scans were performed on two scanners, General Electric (GE) 3Tesla and Siemens 1.5Tesla: the same patient using the same scanner throughout the trial. High-resolution (voxel size GE scanner 0.976x0.976x1.1mm Siemens scanner 1.25x1.25.1.2mm) 3D (volumetric) T1-weighted acquisitions were acquired using an inversion recovery fast spoiled gradient echo (IR-FSPGR) for the GE scanner and Magnetization prepared 180 degrees Radio-frequency pulses and rapid gradient-echo (MPRAGE) for the Siemens scanner. Dual echo fast/turbo spin echo sequence providing 46x 3mm axial proton density and T2 weighted images were acquired.

All scans were pre-processed using N3 bias correction,[w1-2]to reduce intensity non-uniformity. The scans were then subjected to visual quality control (QC) by expert raters. For all visits where more than one scan was available, the best scan was selected for analysis based on this QC. All selected year-2 scans were segmented using the fully automated Multi-Atlas Propagation and Segmentation (MAPS) method.[w3] The segmented regions and scans were checked by trained operators, who performed two types of edits as required: corrections (in most cases minor) to the automated regions for full anatomical coverage and inclusion of all MS lesions in the brain region. Editing was performed semi-automatically using the Medical Image Display and Analysis Software.[24] The regions were then automatically propagated to the year-1 and screening scans using affine and free-form deformation-based non-rigid registration.[w4] Morphological operations (one erosion, two conditional dilations) were subsequently applied to improve boundary location. For six subjects there was no usable year-2 scan available, so the year-1 scans were segmented using MAPS and propagated to screening. All year-2 and year-1 scans were registered to the screening scans and all year-2 scans were additionally registered to the year-1 scans using affine 12 degrees of freedom registration.[w5] Differential Bias Correction (DBC) was performed to reduce any bias in image intensities between the registered scans.[w6] Each pair was then normalised and the brain Boundary Shift Integral (BSI) was calculated using the K-means Normalisation BSI method.[25] Finally all registrations were visually checked and rated.

Additional web-references:

w1. Sled JG, Zijdenbos AP, Evans AC. A Nonparametric Method for Automatic Correction of Intensity Nonuniformity in MRI Data.IEEE Transactions on Medical Imaging. 1998; 17(1):87-97.

w2. Boyes RG, Gunter JL, Frost C, et al. Intensity non-uniformity correction using N3 on 3-T scanners with multichannel phased array coils.Neuroimage 2008; 39(4):1752-62.

w3. Leung KK, Barnes J, Modat M, et al. Brain MAPS: an automated, accurate and robust brain extraction technique using a template library.Neuroimage 2011;55(3):1091-1108.

w4. Rueckert D, Sonoda LI, Hayes C, Hill DL, Leach MO, Hawkes DJ.Nonrigid registration using free-form deformations: application to breast MR images. IEEE Trans Med Imaging 1999;18:712-21.

w5. Woods RP, Grafton ST, Holmes CJ, Cherry SR, Mazziotta JC. Automated image registration: I. General methods and intrasubject, intramodality validation. Journal of Computer Assisted Tomography. 1998;22:139-52.

w6. Lewis EB, Fox NC. Correction of differential intensity inhomogeneity in longitudinal MR images.Neuroimage. 2004;23:75-83.

Web-appendix 5: Frontal lobe volumetric methodology

The brain of each subject was parcellated into 83 non-overlapping regions (Hammers atlas - http://brain-development.org/brain-atlases/individual-adult-brain-atlases-30/) using a multi-atlas propagation and fusion segmentation approach described in [Cardoso et al. Med Image Anal. 2013]. This segmentation process involves the non-linear registration of 30 atlas images, part of the Hammers atlas, to each MS-STAT dataset using the open-source nifty-reg software package with default parameters (https://sourceforge.net/projects/niftyreg/). The obtained non-linear transformations were then used to propagate the manual segmentations associated with each atlas, resulting in 30 candidate segmentations for each MS-STAT dataset. These propagated candidate segmentations were finally fused according to the locally normalised cross correlation between each propagated atlas image and each target image using the parameters suggested in [Cardoso et al. Med Image Anal. 2013] and the nifty-seg open source implementation (https://sourceforge.net/projects/niftyseg/). This process results in a parcellation of each of the MS-Stat brains into 83 non-overlapping regions. Due to the non-overlapping nature of these regions, the frontal volume was finally estimated by simply summing of the volumes of 24 frontal regions as defined in the Hammers protocol, i.e. middle frontal gyrus L, middle frontal gyrus R, precentral gyrus L, precentral gyrus R, straight gyrus L, straight gyrus R, anterior orbital gyrus L, anterior orbital gyrus R, inferior frontal gyrus L, inferior frontal gyrus R, superior frontal gyrus L, superior frontal gyrus R, medial orbital gyrus L, medial orbital gyrus R, lateral orbital gyrus L, lateral orbital gyrus R, posterior orbital gyrus L, posterior orbital gyrus R, subgenual frontal cortex L, subgenual frontal cortex R, subcallosal area L, subcallosal area R, pre-subgenual frontal cortex L, pre-subgenual frontal cortex R. This aggregate frontal region is represented bellow on a randomly chosen subject by the yellow color.

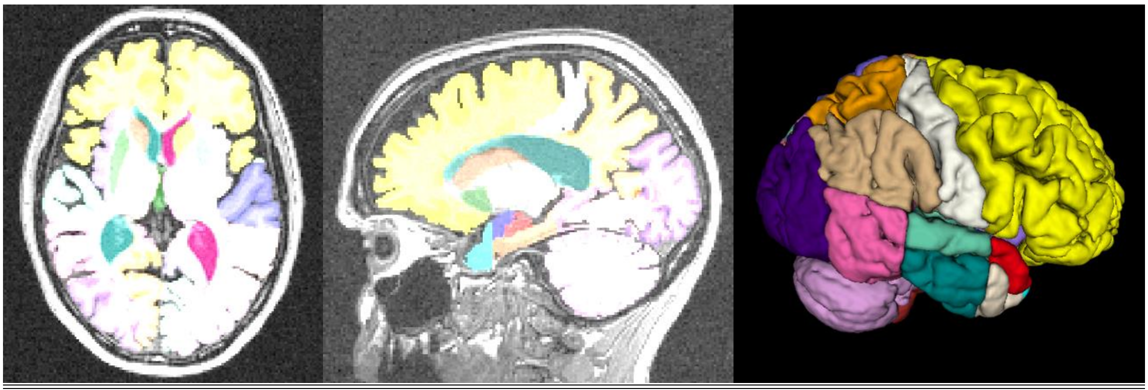

Web appendix 6: additional information for figure 2

% and number of participants with impairment in each domain at baseline

**Measure**	**% (number) impaired in measure (performing >1.5SD below published means)**
WASI IQ	2% (3/130)
WASI Verbal IQ	2% (3/131)
WASI Performance IQ	5% (6/130)
WASI Vocab (verbal intelligence)	4% (5/130)
WASI Similarities (abstract verbal reasoning)	5% (7/129)
WASI block (spatial perception, visuomotor skills)	6% (8/129)
WASI matrix (non-verbal abstract reasoning)	8% (10/129)
GNT (semantic memory)	11% (14/130)
BMIPB story immediate (verbal recall)	23% (30/131)
BMIPB story delayed (verbal recall)	21% (28/131)
BMIPB figure copying	12% (15/130)
BMIPB figure immediate (non-verbal recall)	33% (43/130)
BMIPB figure delayed (non-verbal recall)	10% (13/130)
VOSP cube analysis (spatial perception)	11% (15/132)
FAB (executive function)	45% (60/133)
PASAT	46% (62/134)
HRQL MCS	12% (15/122)
HRQL PCS	56% (68/122)
HRQL physical functioning	84% (109/130)
HRQL role limitations physical	52% (67/130)
HRQL bodily pain	13% (18/135)
HRQL general health	37% (48/131)
HRQL vitality	21% (27/130)
HRQL social functioning	28% (38/134)
HRQL role limitations emotional	37% (49/133)
HRQL mental health	13% (17/131)

Web appendix 7: Mean and individual patient values for change between baseline and 2 years for the FAB and HRQL outcomes. Means are indicated by solid bars; individual points show patient values. A positive value indicates an improvement in that outcome.

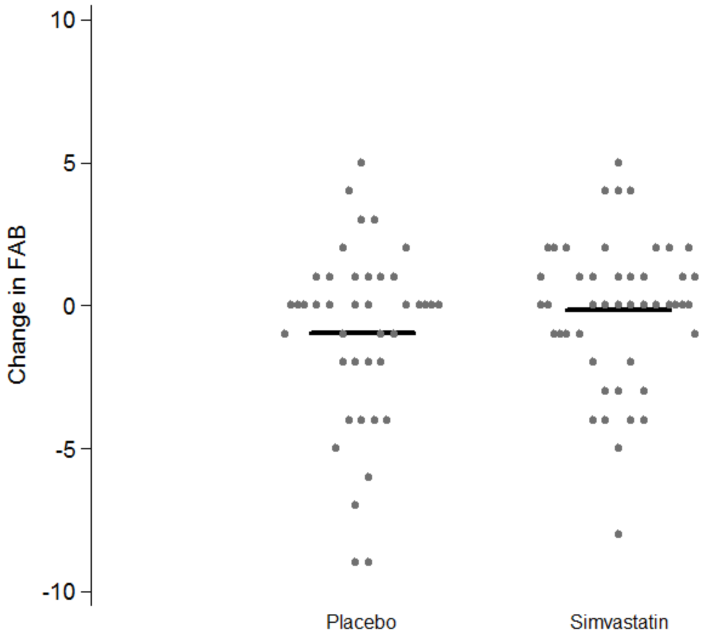

Figure 7A: Frontal Assessment Battery (FAB) (executive function)

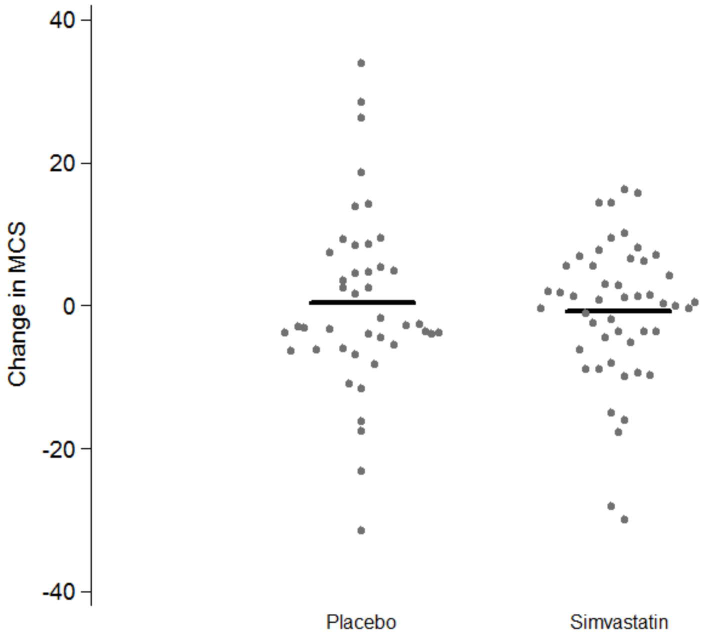

Figure 7B: Health related quality of life (HRQL) Mental Component Score (MCS)

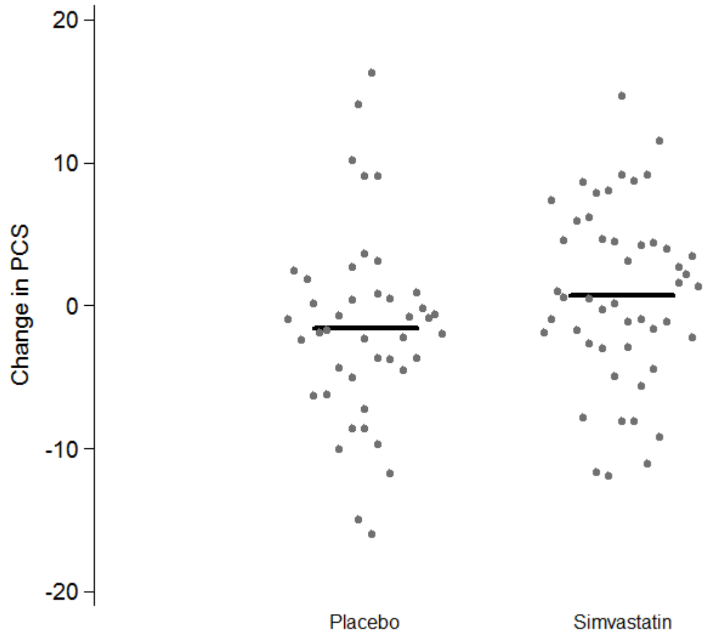

Figure 7C: health related quality of life (HRQL) Physical Component Score (PCS)
